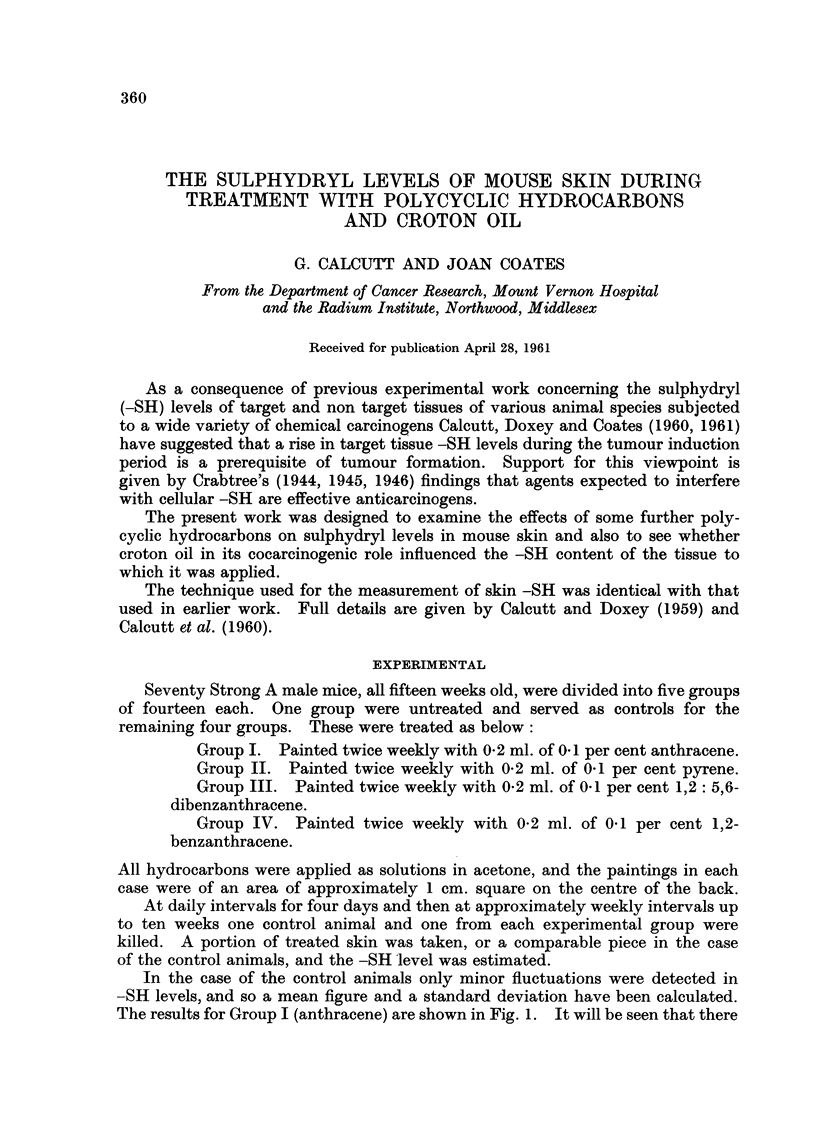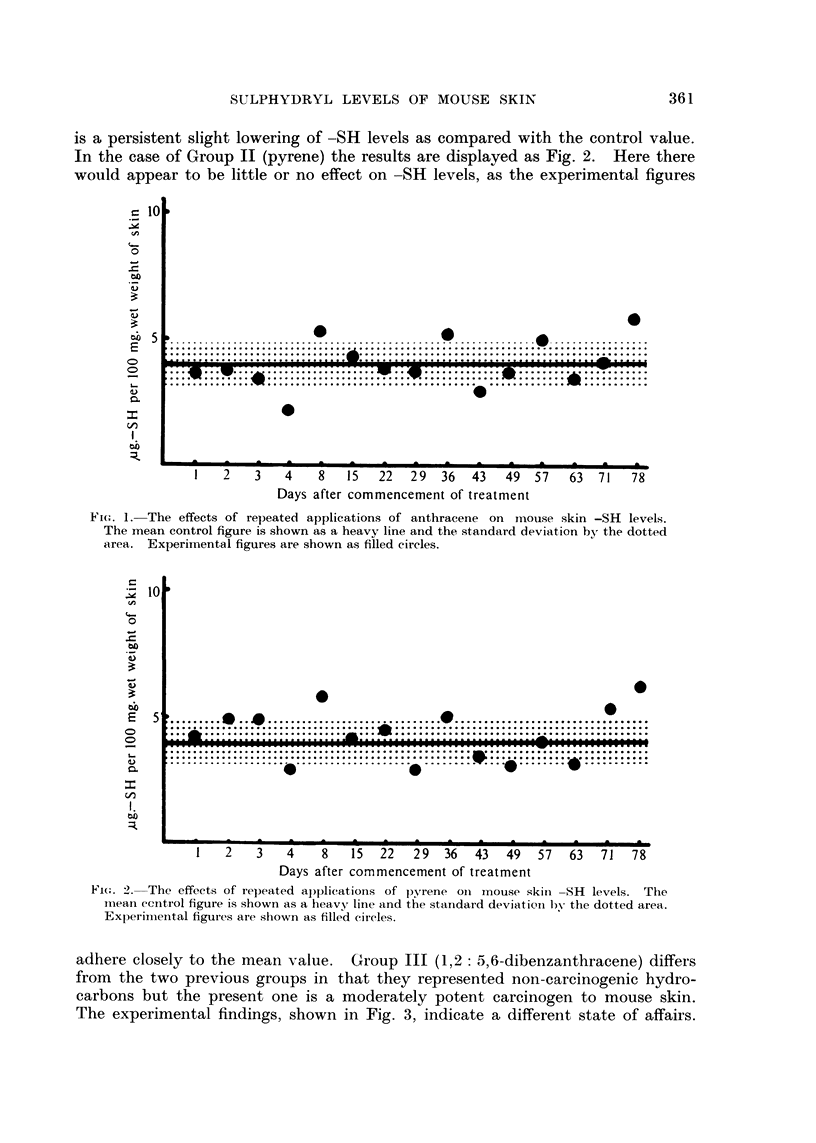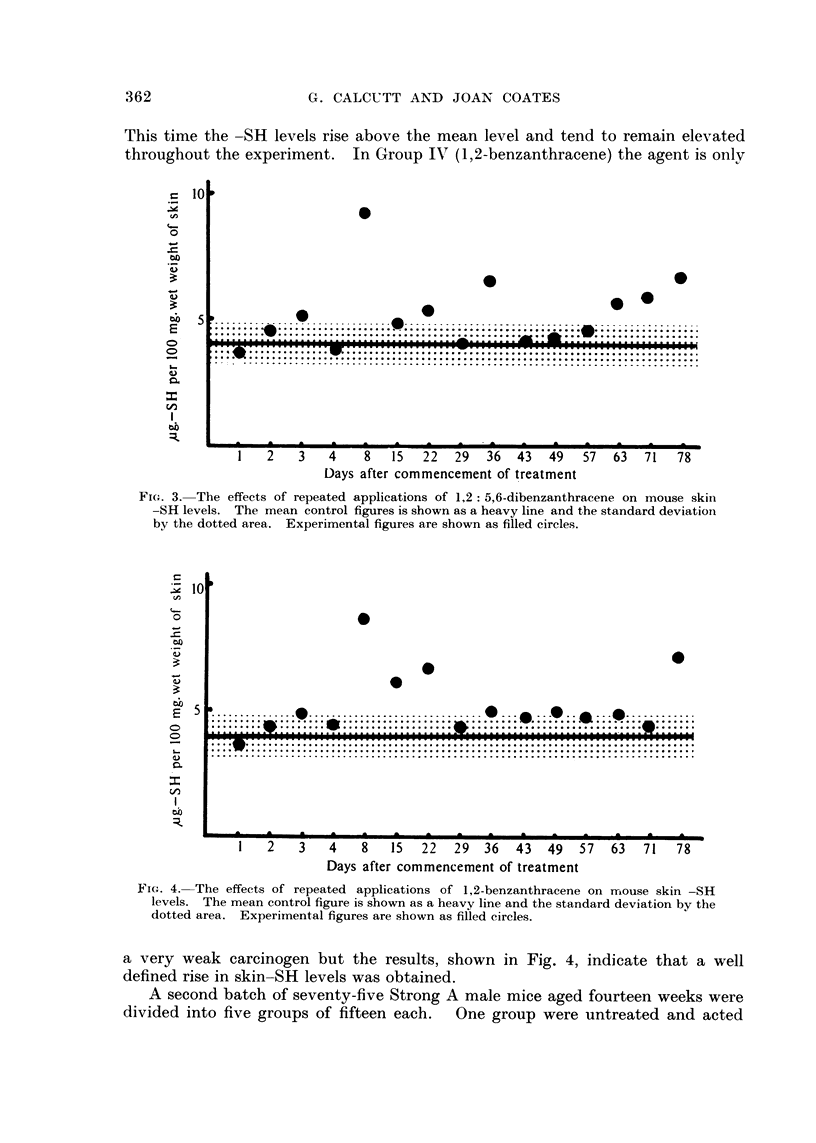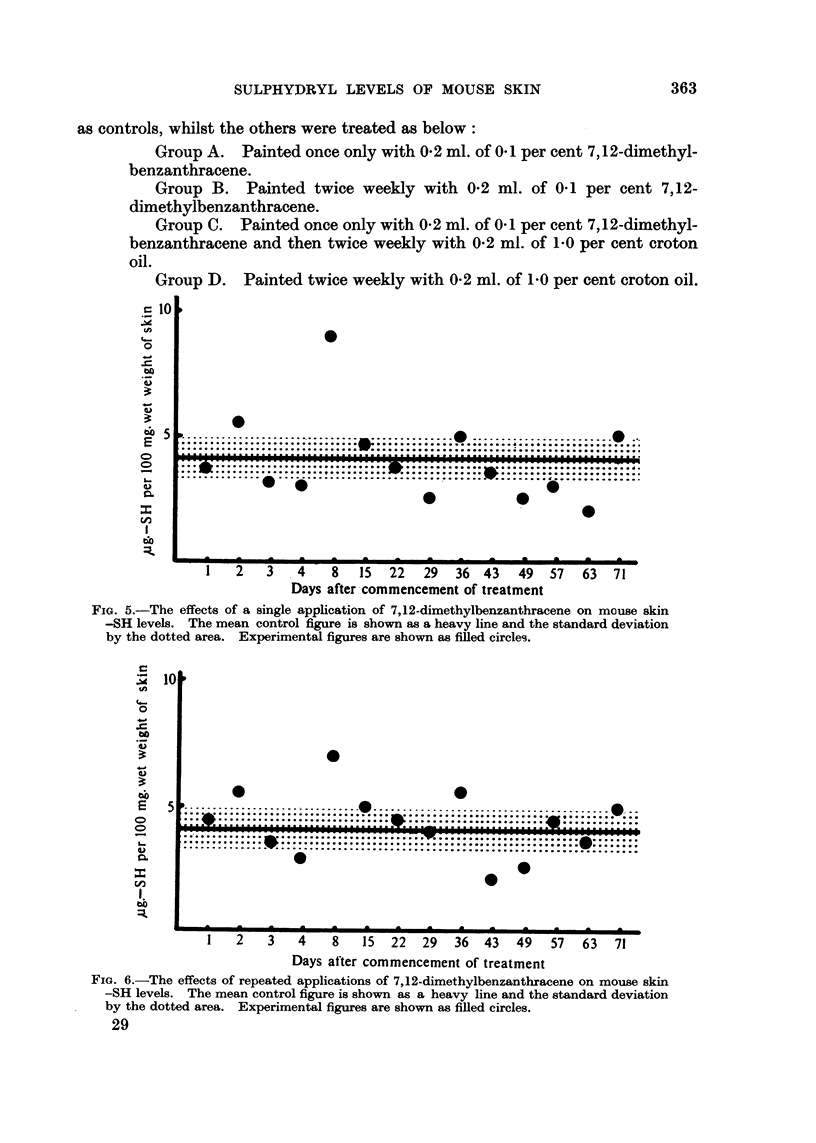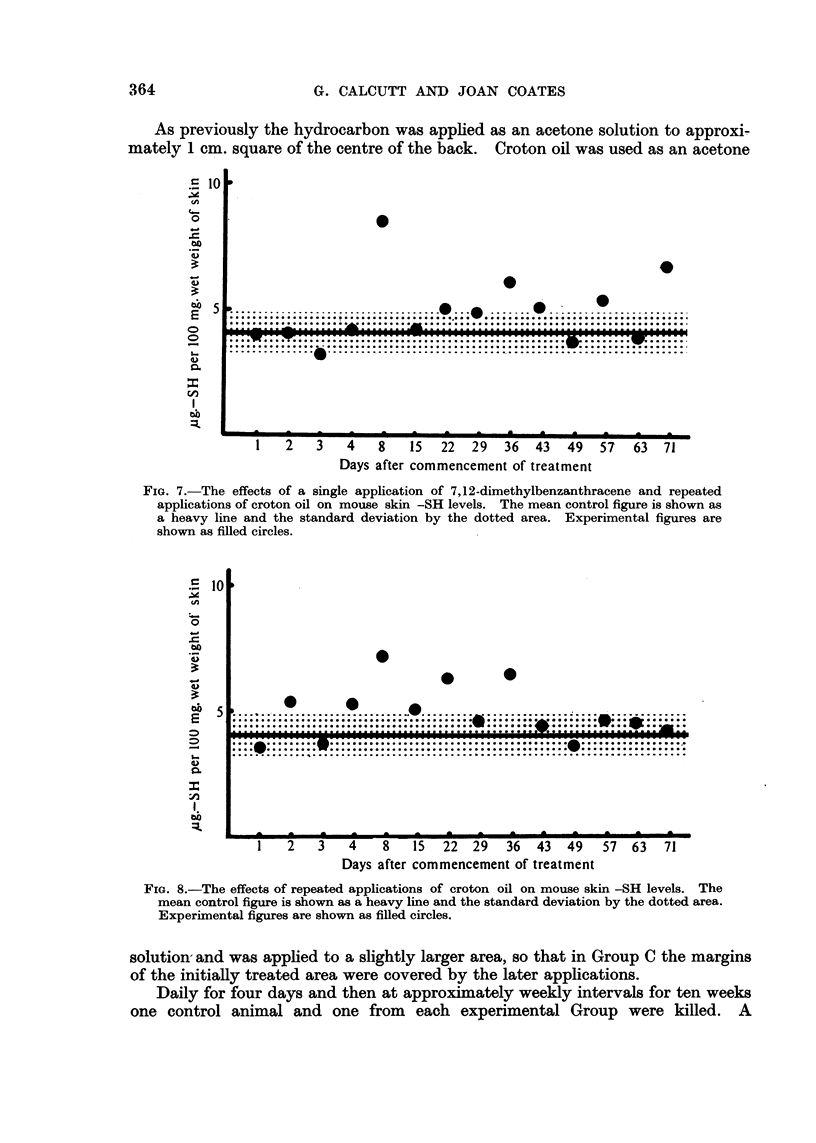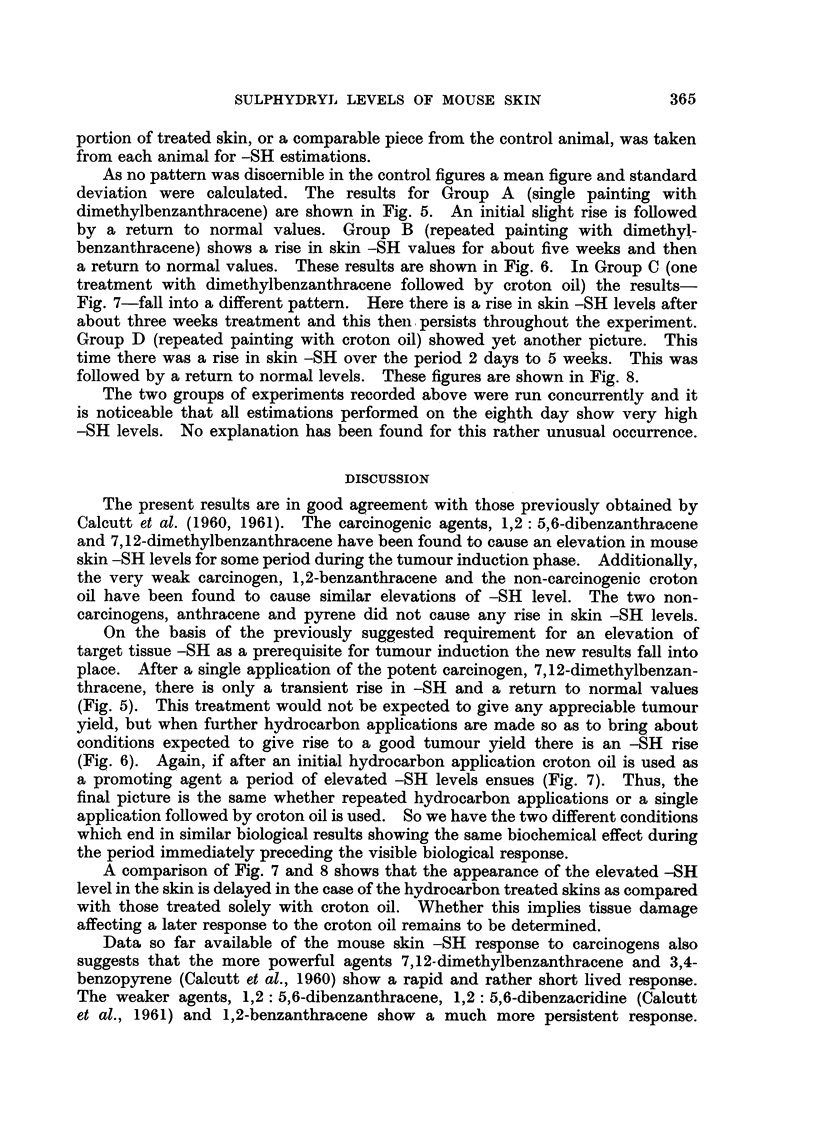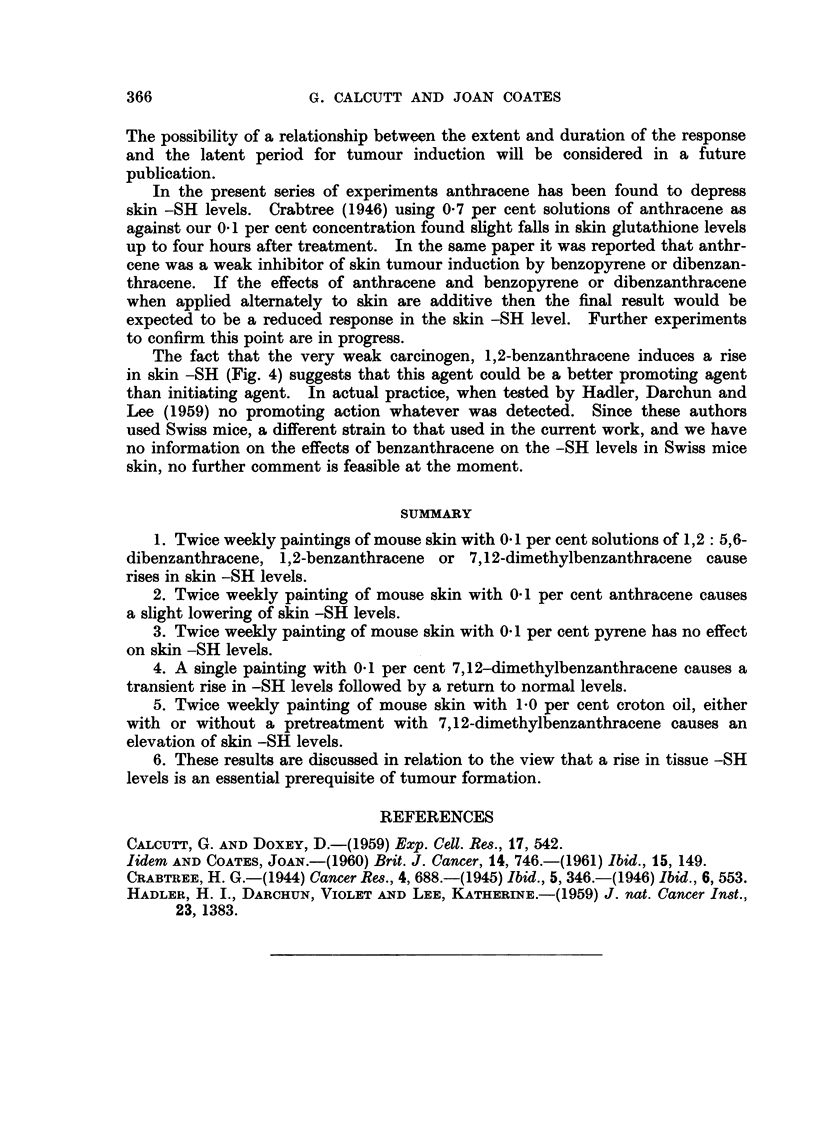# The Sulphydryl Levels of Mouse Skin During Treatment with Polycyclic Hydrocarbons and Croton Oil

**Published:** 1961-06

**Authors:** G. Calcutt, Joan Coates


### THE SULPHYDRYL LEVELS OF MOUSE SKIN DURING TREATMENT WITH POLYCYCLIC HYDROCARBONS AND CROTON OIL

G. CALCUTT AND JOAN COATES

*From the Department of Cancer Research, Mount Vernon Hospital and the Radium Institute, Northwood, Middlesex*

Received for publication April 28, 1961

As a consequence of previous experimental work concerning the sulphydryl (–SH) levels of target and non target tissues of various animal species subjected to a wide variety of chemical carcinogens Calcutt, Doxey and Coates (1960, 1961) have suggested that a rise in target tissue –SH levels during the tumour induction period is a prerequisite of tumour formation. Support for this viewpoint is given by Crabtree's (1944, 1945, 1946) findings that agents expected to interfere with cellular –SH are effective anticarcinogens.

The present work was designed to examine the effects of some further polycyclic hydrocarbons on sulphydryl levels in mouse skin and also to see whether croton oil in its cocarcinogenic role influenced the –SH content of the tissue to which it was applied.

The technique used for the measurement of skin –SH was identical with that used in earlier work. Full details are given by Calcutt and Doxey (1959) and Calcutt *et al.* (1960).

### EXPERIMENTAL

Seventy Strong A male mice, all fifteen weeks old, were divided into five groups of fourteen each. One group were untreated and served as controls for the remaining four groups. These were treated as below :

Group I. Painted twice weekly with 0·2 ml. of 0·1 per cent anthracene.

Group II. Painted twice weekly with 0·2 ml. of 0·1 per cent pyrene.

Group III. Painted twice weekly with 0·2 ml. of 0·1 per cent 1,2 : 5,6-dibenzanthracene.

Group IV. Painted twice weekly with 0·2 ml. of 0·1 per cent 1,2-benzanthracene.

All hydrocarbons were applied as solutions in acetone, and the paintings in each case were of an area of approximately 1 cm. square on the centre of the back.

At daily intervals for four days and then at approximately weekly intervals up to ten weeks one control animal and one from each experimental group were killed. A portion of treated skin was taken, or a comparable piece in the case of the control animals, and the –SH level was estimated.

In the case of the control animals only minor fluctuations were detected in –SH levels, and so a mean figure and a standard deviation have been calculated. The results for Group I (anthracene) are shown in Fig. 1. It will be seen that there is a persistent slight lowering of –SH levels as compared with the control value. In the case of Group II (pyrene) the results are displayed as Fig. 2. Here there would appear to be little or no effect on –SH levels, as the experimental figures

FIG. 1.—The effects of repeated applications of anthracene on mouse skin –SH levels. The mean control figure is shown as a heavy line and the standard deviation by the dotted area. Experimental figures are shown as filled circles.

FIG. 2.—The effects of repeated applications of pyrene on mouse skin –SH levels. The mean control figure is shown as a heavy line and the standard deviation by the dotted area. Experimental figures are shown as filled circles.

adhere closely to the mean value. Group III (1,2 : 5,6-dibenzanthracene) differs from the two previous groups in that they represented non-carcinogenic hydrocarbons but the present one is a moderately potent carcinogen to mouse skin. The experimental findings, shown in Fig. 3, indicate a different state of affairs.

This time the –SH levels rise above the mean level and tend to remain elevated throughout the experiment. In Group IV (1,2-benzanthracene) the agent is only

Fig. 3.—The effects of repeated applications of 1,2 : 5,6-dibenzanthracene on mouse skin –SH levels. The mean control figures is shown as a heavy line and the standard deviation by the dotted area. Experimental figures are shown as filled circles.

Fig. 4.—The effects of repeated applications of 1,2-benzanthracene on mouse skin –SH levels. The mean control figure is shown as a heavy line and the standard deviation by the dotted area. Experimental figures are shown as filled circles.

a very weak carcinogen but the results, shown in Fig. 4, indicate that a well defined rise in skin–SH levels was obtained.

A second batch of seventy-five Strong A male mice aged fourteen weeks were divided into five groups of fifteen each. One group were untreated and acted as controls, whilst the others were treated as below :

Group A. Painted once only with 0·2 ml. of 0·1 per cent 7,12-dimethylbenzanthracene.

Group B. Painted twice weekly with 0·2 ml. of 0·1 per cent 7,12-dimethylbenzanthracene.

Group C. Painted once only with 0·2 ml. of 0·1 per cent 7,12-dimethylbenzanthracene and then twice weekly with 0·2 ml. of 1·0 per cent croton oil.

Group D. Painted twice weekly with 0·2 ml. of 1·0 per cent croton oil.

Fig. 5.—The effects of a single application of 7,12-dimethylbenzanthracene on mouse skin –SH levels. The mean control figure is shown as a heavy line and the standard deviation by the dotted area. Experimental figures are shown as filled circles.

Fig. 6.—The effects of repeated applications of 7,12-dimethylbenzanthracene on mouse skin –SH levels. The mean control figure is shown as a heavy line and the standard deviation by the dotted area. Experimental figures are shown as filled circles.

As previously the hydrocarbon was applied as an acetone solution to approximately 1 cm. square of the centre of the back. Croton oil was used as an acetone

FIG. 7.—The effects of a single application of 7,12-dimethylbenzanthracene and repeated applications of croton oil on mouse skin –SH levels. The mean control figure is shown as a heavy line and the standard deviation by the dotted area. Experimental figures are shown as filled circles.

FIG. 8.—The effects of repeated applications of croton oil on mouse skin –SH levels. The mean control figure is shown as a heavy line and the standard deviation by the dotted area. Experimental figures are shown as filled circles.

solution and was applied to a slightly larger area, so that in Group C the margins of the initially treated area were covered by the later applications.

Daily for four days and then at approximately weekly intervals for ten weeks one control animal and one from each experimental Group were killed. A

portion of treated skin, or a comparable piece from the control animal, was taken from each animal for –SH estimations.

As no pattern was discernible in the control figures a mean figure and standard deviation were calculated. The results for Group A (single painting with dimethylbenzanthracene) are shown in Fig. 5. An initial slight rise is followed by a return to normal values. Group B (repeated painting with dimethylbenzanthracene) shows a rise in skin –SH values for about five weeks and then a return to normal values. These results are shown in Fig. 6. In Group C (one treatment with dimethylbenzanthracene followed by croton oil) the results—Fig. 7—fall into a different pattern. Here there is a rise in skin –SH levels after about three weeks treatment and this then persists throughout the experiment. Group D (repeated painting with croton oil) showed yet another picture. This time there was a rise in skin –SH over the period 2 days to 5 weeks. This was followed by a return to normal levels. These figures are shown in Fig. 8.

The two groups of experiments recorded above were run concurrently and it is noticeable that all estimations performed on the eighth day show very high –SH levels. No explanation has been found for this rather unusual occurrence.

### DISCUSSION

The present results are in good agreement with those previously obtained by Calcutt *et al.* (1960, 1961). The carcinogenic agents, 1,2 : 5,6-dibenzanthracene and 7,12-dimethylbenzanthracene have been found to cause an elevation in mouse skin –SH levels for some period during the tumour induction phase. Additionally, the very weak carcinogen, 1,2-benzanthracene and the non-carcinogenic croton oil have been found to cause similar elevations of –SH level. The two non-carcinogens, anthracene and pyrene did not cause any rise in skin –SH levels.

On the basis of the previously suggested requirement for an elevation of target tissue –SH as a prerequisite for tumour induction the new results fall into place. After a single application of the potent carcinogen, 7,12-dimethylbenzanthracene, there is only a transient rise in –SH and a return to normal values (Fig. 5). This treatment would not be expected to give any appreciable tumour yield, but when further hydrocarbon applications are made so as to bring about conditions expected to give rise to a good tumour yield there is an –SH rise (Fig. 6). Again, if after an initial hydrocarbon application croton oil is used as a promoting agent a period of elevated –SH levels ensues (Fig. 7). Thus, the final picture is the same whether repeated hydrocarbon applications or a single application followed by croton oil is used. So we have the two different conditions which end in similar biological results showing the same biochemical effect during the period immediately preceding the visible biological response.

A comparison of Fig. 7 and 8 shows that the appearance of the elevated –SH level in the skin is delayed in the case of the hydrocarbon treated skins as compared with those treated solely with croton oil. Whether this implies tissue damage affecting a later response to the croton oil remains to be determined.

Data so far available of the mouse skin –SH response to carcinogens also suggests that the more powerful agents 7,12-dimethylbenzanthracene and 3,4-benzopyrene (Calcutt *et al.*, 1960) show a rapid and rather short lived response. The weaker agents, 1,2 : 5,6-dibenzanthracene, 1,2 : 5,6-dibenzacridine (Calcutt *et al.*, 1961) and 1,2-benzanthracene show a much more persistent response.

The possibility of a relationship between the extent and duration of the response and the latent period for tumour induction will be considered in a future publication.

In the present series of experiments anthracene has been found to depress skin –SH levels. Crabtree (1946) using 0·7 per cent solutions of anthracene as against our 0·1 per cent concentration found slight falls in skin glutathione levels up to four hours after treatment. In the same paper it was reported that anthrcene was a weak inhibitor of skin tumour induction by benzopyrene or dibenzanthracene. If the effects of anthracene and benzopyrene or dibenzanthracene when applied alternately to skin are additive then the final result would be expected to be a reduced response in the skin –SH level. Further experiments to confirm this point are in progress.

The fact that the very weak carcinogen, 1,2-benzanthracene induces a rise in skin –SH (Fig. 4) suggests that this agent could be a better promoting agent than initiating agent. In actual practice, when tested by Hadler, Darchun and Lee (1959) no promoting action whatever was detected. Since these authors used Swiss mice, a different strain to that used in the current work, and we have no information on the effects of benzanthracene on the –SH levels in Swiss mice skin, no further comment is feasible at the moment.

### SUMMARY

1. Twice weekly paintings of mouse skin with 0·1 per cent solutions of 1,2 : 5,6-dibenzanthracene, 1,2-benzanthracene or 7,12-dimethylbenzanthracene cause rises in skin –SH levels.
2. Twice weekly painting of mouse skin with 0·1 per cent anthracene causes a slight lowering of skin –SH levels.
3. Twice weekly painting of mouse skin with 0·1 per cent pyrene has no effect on skin –SH levels.
4. A single painting with 0·1 per cent 7,12–dimethylbenzanthracene causes a transient rise in –SH levels followed by a return to normal levels.
5. Twice weekly painting of mouse skin with 1·0 per cent croton oil, either with or without a pretreatment with 7,12-dimethylbenzanthracene causes an elevation of skin –SH levels.
6. These results are discussed in relation to the view that a rise in tissue –SH levels is an essential prerequisite of tumour formation.

### REFERENCES

Calcutt, G. and Doxey, D.—(1959) *Exp. Cell. Res.*, **17**, 542.

*Iidem* and Coates, Joan.—(1960) *Brit. J. Cancer*, **14**, 746.—(1961) *Ibid.*, **15**, 149.

Crabtree, H. G.—(1944) *Cancer Res.*, **4**, 688.—(1945) *Ibid.*, **5**, 346.—(1946) *Ibid.*, **6**, 553.

Hadler, H. I., Darchun, Violet and Lee, Katherine.—(1959) *J. nat. Cancer Inst.*, **23**, 1383.